# What to Eat

**Published:** 1870-07

**Authors:** 


### WHAT TO EAT.

People are too apt to render themselves liable to disease by means of the food taken into the stomach.

During hot weather like the present, the system is best supported by a vegetable diet.—Very little, or no meat at all should be eaten, as the temperature of the body is greatly increased through its instrumentality, while the opposite effect should be obtained. Health is best preserved in those who live upon mixed diet. It is not a good plan to have the same meats and the same vegetables every day for a week—change them frequently. Without fresh vegetables we would soon become diseased with scurvey. The vegetables possessing the highest proportion of flesh-forming principle are the bean, pea, beet, &c.

Two meals a day is best in hot weather.—Breakfast at about nine in the morning and supper at four in the afternoon. You then escape the greatest heat of the day, when it is absolutely injurious to eat plentifully.

Milk should not be drunk at meals during hot weather, it will render you billious.

In fact, very little drink of any kind should be indulged in at meals. A wine glass of sour wine—pure grape juice—is the most health producing drink at meals—it assists digestion, when the secretions in the stomach are not as plentifully supplied as in cool weather.

Give more of your attenaion to your diet this summer and learn whether you can not improve your health thereby.

### FLIES ON HORSES.

The following is given as a preventative of horses being teased by flies: Take two or three small handfuls of walnut leaves, upon which pour two or three quarts of cold water; let it infuse one night, and pour the whole into a kettle and let it boil for a quarter of an hour. When cold it will be fit for use. No more is required than to moisten a sponge, and before the horse goes out of the stable, let those parts that are most irritable be smeared over with the liquor, viz: between and upon the ears, the neck, the flanks, etc. Not only the gentleman or lady who rides out for pleasure, will derive pleasure from the walnut leaves thus prepared, but the coachman, the wagoner, and all others who use horses during the hot months.

